# 3D observational analysis of convection around and inside a self-propelled droplet†

**DOI:** 10.1039/d4ra09004g

**Published:** 2025-05-07

**Authors:** Tamako Suzuki, Hideyuki Sawada

**Affiliations:** a Department of Pure and Applied Physics, Graduated School of Advanced Science and Engineering, Waseda University 3-4-1 Okubo, Shinjuku-Ku Tokyo 169-8555 Japan tamakapi_omc53@toki.waseda.jp; b Faculty of Science and Engineering, Waseda University 3-4-1 Okubo, Shinjuku-Ku Tokyo 169-8555 Japan

## Abstract

This study aims to analyze the convection flow generated three-dimensionally around and inside a 1-pentanol droplet dropped into a 1-pentanol aqueous solution. The difference in concentration between the droplet and the aqueous solution causes an interfacial tension gradient, and then the droplet starts moving in the aqueous solution. The droplet shape is closely related to its self-propulsion behavior because the interfacial tension gradient changes with the droplet shape. In this study, we fix the droplet shape using an exoskeleton to control the self-propulsion direction. The exoskeleton is fabricated by using OHP film with a circular-shape having a hole in the center, and a droplet is dropped into the hole to fix the droplet shape. We prepared two different exoskeletons having symmetrical elliptical holes and asymmetrical elliptical holes. We also prepare two different concentrations of aqueous solutions. By using two different concentrations of aqueous solution and two types of exoskeletons, we analyze the behavior of the droplet dropped into the exoskeleton hole, in relation with the convection around and inside the droplet. The results indicate that the self-propulsion direction of the droplet is determined by the shape of the droplet, which is fixed by the exoskeleton. Particularly in the case of the asymmetrical exoskeleton, the self-propulsion direction is fixed in one direction. The self-propulsion velocity of the droplets changed depending on the concentration of the aqueous solution, and we observed the droplet to self-propel several times per 50 seconds when the aqueous solution of smaller concentration was used. Based on these experimental results, we discuss the dominant factors to determine the self-propulsion direction by visualizing the convection around and inside the droplet.

## Abstract 2

This paper discusses the dominant factors to determine the self-propulsion direction by visualizing the convection around and inside the droplet.

## Graphical abstract

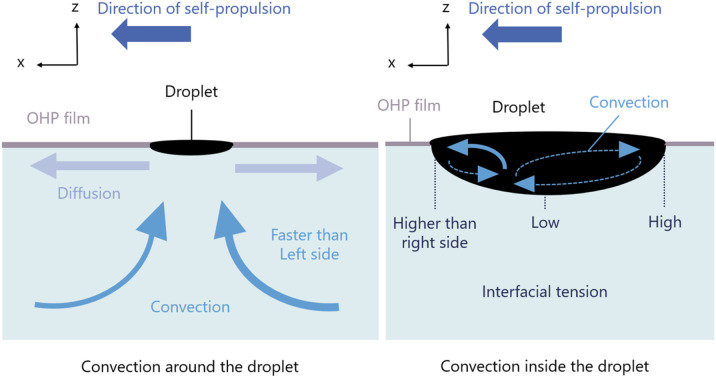

## 1 Introduction

The study of self-propulsion caused by the Marangoni effect has attracted much interest.^1–6^ The Marangoni effect is a phenomenon in which a difference in interfacial tension is generated due to a difference in temperature or concentration, and the flow is generated by pulling toward the direction of higher interfacial tension. In addition to temperature and concentration, other parameters such as droplet volume, the depth of aqueous solution, and chemical reactions depending on the material are complexly involved in this phenomenon.^7^

Marangoni surfers, which are self-propelled by the Marangoni effect, include camphor,^8–12^ gels,^13–15^ and droplets.^16–27^ The most studied type is the camphor. The camphor floating on water releases a surfactant, causing a difference in interfacial tension, and starts self-propulsion toward the direction of higher interfacial tension. Examples of self-propelled gels include calcium alginate^28,29^ and PNIPAM-based microgel.^30–32^ These gels shrink by expelling the solvent from the gel, and the shape of the gel is determined. Due to the asymmetric interfacial tension around the gel caused by its shape, the gel begins to self-propel in the direction of higher interfacial tension. Examples of self-propelled droplets include 1-pentanol,^33–35^ anhydrous oleic acid,^36–38^ and aniline.^39^ In this study, we conduct experimental studies on self-propelled droplets by using the 1-pentanol droplet and the 1-pentanol aqueous solution. We study this system because self-propulsion occurs due to a physical factor: the difference in concentration between the droplet and the aqueous solution.

We pay attention to the Marangoni convection generated around and inside a droplet. No studies are found that have actually observed the convection generated around and inside the droplet to be associated with the droplet's self-propulsion. In this study, we examine the causes that affect droplet self-propulsion by observing convection in three dimensions. To control the direction of self-propulsion, it is necessary to intentionally create the interfacial tension difference, which causes the Marangoni effect. In our previous studies, we introduced an exoskeleton for the directional and velocity control of anhydrous oleic acid oil droplets.^36^ A droplet shaped as a boomerang by an exoskeleton locomoted in the direction from a concave region to a convex region. When a hole had an asymmetrical boomerang shape the droplet made a circular motion in the direction of the larger width. We have applied to a transporting robot driven only by the energy obtained from chemical reactions based on these results.

To control the direction of self-propulsion, we pay attention to the Marangoni convection generated around and inside a droplet. It is necessary to intentionally create the interfacial tension difference, which causes the Marangoni effect. In our previous studies, we introduced an exoskeleton for the directional and velocity control of anhydrous oleic acid oil droplets.^36^ A droplet shaped as a boomerang by an exoskeleton locomoted in the direction from a concave region to a convex region. When a hole had an asymmetrical boomerang shape, the droplet made a circular motion in the direction of the larger width.

In this study, we also use the exoskeleton to control the direction of the self-propelled droplet. When we use the system of the 1-pentanol droplet and the 1-pentanol aqueous solution, we fabricate the exoskeleton with the hole of an elongated elliptical shape, and the 1-pentanol droplet is fixed to that shape by dropping it into the exoskeleton. The difference in curvature between the end edge and the center of the droplet causes a difference in the diffusion rate of 1-pentanol, resulting in a difference in concentration. At this time, a difference in concentration appears between both end edges spontaneously. This asymmetry causes the droplet to self-propel in the direction of the extended major axis of the ellipse. This result indicates that using an exoskeleton is useful to determine the direction of self-propulsion. In addition, the exoskeleton also enables the droplet to fix its position, which allows us to observe the Marangoni convection generated around and inside the droplet. We find that the superposition of the Marangoni convection generated around and inside the droplet induced the self-propulsion of the droplet.

This study focuses on the convection generated around and inside a 1-pentanol droplet dropped into the exoskeleton, which floats in the aqueous solution. Firstly, we observe the changes in the self-propulsion behavior of the droplet depending on the exoskeleton and the concentration of the aqueous solution. Then, we observe the three-dimensional convection generated around and inside the droplet from the two directions. We analyze the relation between the convection and the self-propulsion to understand the dominant factor determining the self-propulsion direction based on these experimental results.

## 2 Materials

We prepare a 1-pentanol aqueous solution, mixed with 1-pentanol (FUJIFILM) and pure water, and stir with a magnetic stirrer (AS ONE, RS-6AN) for 1 hour. The concentration of the aqueous solution is indicated in each section of the experimental method. A droplet of 1-pentanol only is dropped into the aqueous solution.

## 3 Preliminary experiment

### 3.1 Experimental method

**Fig. 1:**
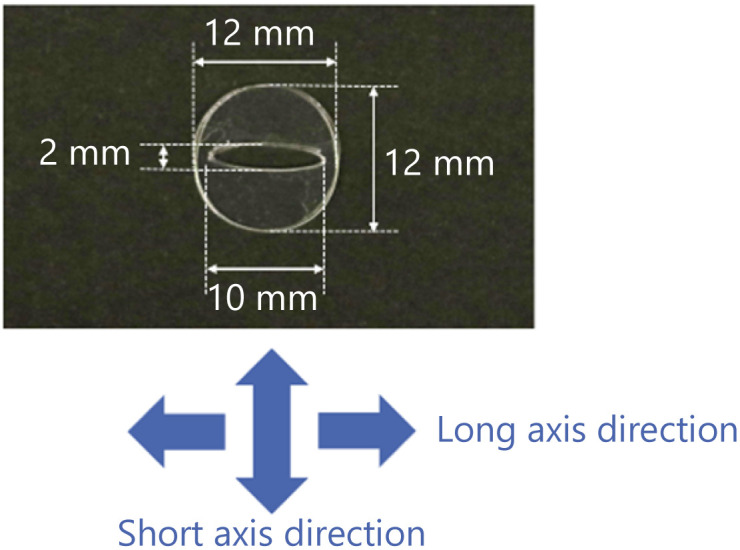
The exoskeleton used in the preliminary experiment.

We fabricate an exoskeleton to fix the droplet shape as shown in Fig. 1. The exoskeleton is designed as a 12 mm diameter circular shape with an elongated elliptical hole in the center. The exoskeleton is made of a 0.10 mm-thick overhead projector (OHP, polyester) film. A sheet of an OHP film is cut by a cutting plotter (Brother, SDX85), and two OHP films are bonded using instant glue to make an exoskeleton with a thickness of 0.20 mm. The arrows at the bottom of Fig. 1 indicate the directions relative to the exoskeleton to show the self-propulsion direction.

**Fig. 2:**
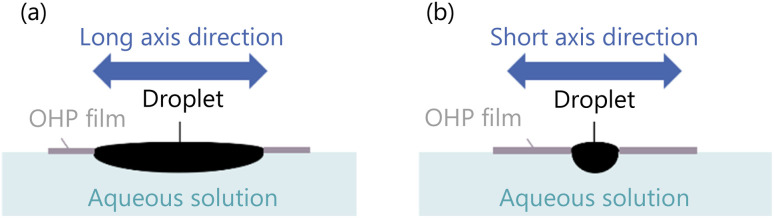
The shape of the droplet dropped into an exoskeleton. (a) The droplet shape in the long axis direction. (b) The droplet shape in the short axis direction.

We drop a 6 μL 1-pentanol droplet into the hole of the exoskeleton. The shape of the hole in the exoskeleton, and the droplet volume are determined so that the droplet does not overflow through the hole. We confirm that the droplet shape is properly fixed as shown in Fig. 2 without overflowing from the hole. As discussed in our previous studies,^35^ a droplet dropped into the exoskeleton self-propels in the long axis direction.

**Fig. 3:**
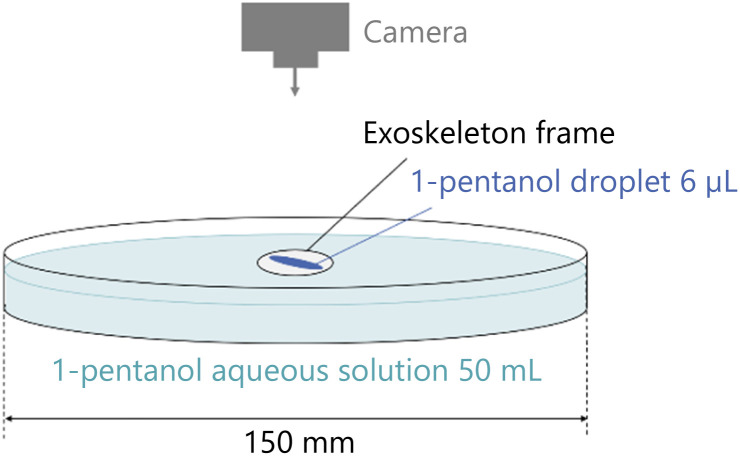
Experimental setup of the preliminary experiment.

Firstly, we conduct an experiment to measure the self-propulsion velocity of a droplet dropped into the exoskeleton to properly determine the concentration of the aqueous solution used in this study. The experimental setup is shown in Fig. 3. After filling 50 mL of 1-pentanol aqueous solution in a Petri dish with a diameter of 150 mm, we float an exoskeleton on the aqueous solution. Then, we introduce a 6 μL of 1-pentanol droplet in the exoskeleton using an automatic micropipette. The behavior of the self-propelled droplet is captured using a single-lens reflex camera (Canon, EOS 80D, 1280 × 720 px, 270 × 152 mm, 29.97 fps) placed directly above the Petri dish. The movies are analyzed using Kinovea software. The experiments are performed on an experimental desk placed horizontally. The temperature is about 25 °C and the humidity is about 60%.

We prepare six different concentrations of 1-pentanol aqueous solution. We mix 0.5, 1.0, 1.5, 2.0, 2.3, and 2.5 mL of 1-pentanol with 100 mL of pure water to not exceed the saturation concentration. We conduct three trials for each of the six different concentrations of aqueous solutions.

### 3.2 Experimental result

**Fig. 4:**
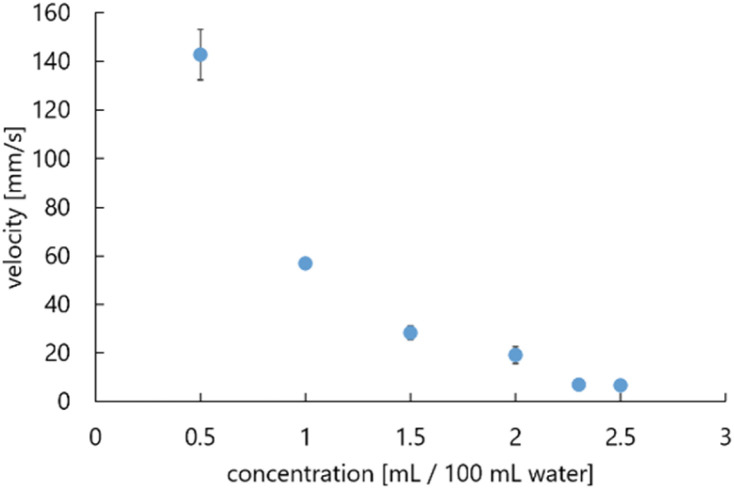
Relationship between concentration of aqueous solution and average velocities of self-propelled droplets.

In all trials, we found that the droplets were self-propelled in the long axis direction. The average of maximum velocities during self-propulsion and the corresponding standard deviations are shown in Fig. 4. We found a negative correlation between the concentration of 1-pentanol aqueous solution and the average velocities of self-propelled 1-pentanol droplets.

We analyzed the causes of these results by the effect of the convection inside the aqueous solution around the droplet, and by the convection inside the droplet, separately. Firstly, the convection around the droplet is explained by Fick's first law:1
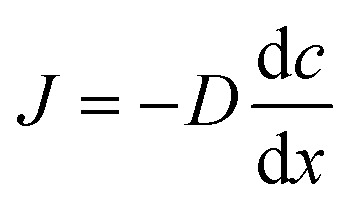
where *J* is the diffusion flux, *D* is the diffusion coefficient, *c* is the concentration, and *x* is the position. We adopted the function of the *x* variable in eqn (1) because the droplet is self-propelled straightly in one direction. Since the diffusion velocity is proportional to the concentration gradient, the convection generated by diffusion becomes greater, and the self-propulsion velocity becomes faster.

**Fig. 5:**
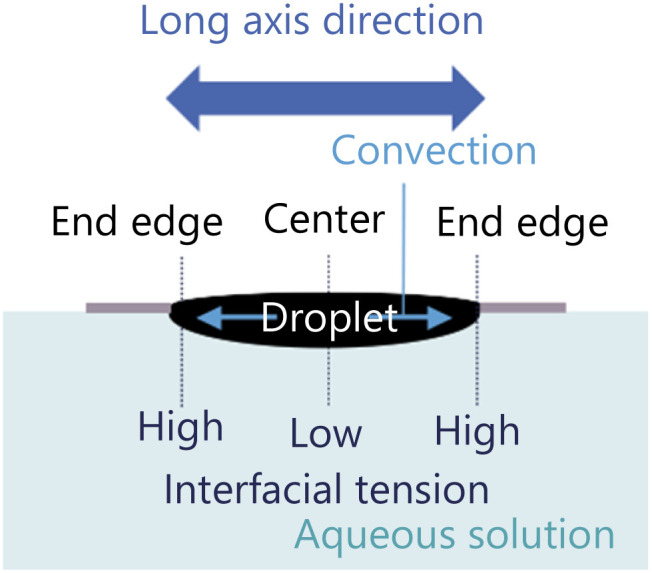
Schematic diagram of the relationship between the interfacial tension and convection inside the droplet.

On the other hand, the convection inside the droplet is explained by the difference in the interfacial tension due to the droplet shape. The droplet is fixed to an elongated elliptical shape by the exoskeleton as shown in Fig. 5. The difference in curvature between the end edge and center of the droplet causes a difference in the diffusion flux of 1-pentanol, resulting in a concentration gradient. This concentration gradient becomes a difference in interfacial tension. The difference in the interfacial tension causes the flow from the center to the end edge. This interfacial tension difference *γ* is expressed as follows:2
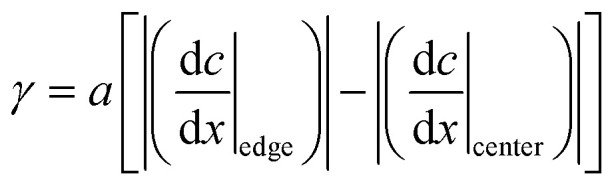
where *a* is a constant, *c* is the concentration of the 1-pentanol solution, *x* is the position, d*c*/d*x*|_edge_ is the concentration gradient at the end edge of the droplet, and d*c*/d*x*|_center_ is the concentration gradient in the center of the droplet. The interfacial tension difference decreases with the 1-pentanol concentration of the aqueous solution. Therefore, the smaller the aqueous solution concentration is, the greater the convection inside the droplet becomes, which results in the increasing velocity of self-propulsion.

Thus, the velocity of the droplet depends on the concentration of the aqueous solution as shown in Fig. 4 Based on this result, we use two types of 1-pentanol aqueous solutions: one with the concentration of 0.5 mL/100 mL water, in which the fastest velocity was measured, and the other with the concentration of 2.3 mL/100 mL water, which was used in our previous study.^35^

## 4 Experiments and analysis

### 4.1 Experiment 1: observation of behavior

#### 4.1.1 Experimental method

**Fig. 6:**
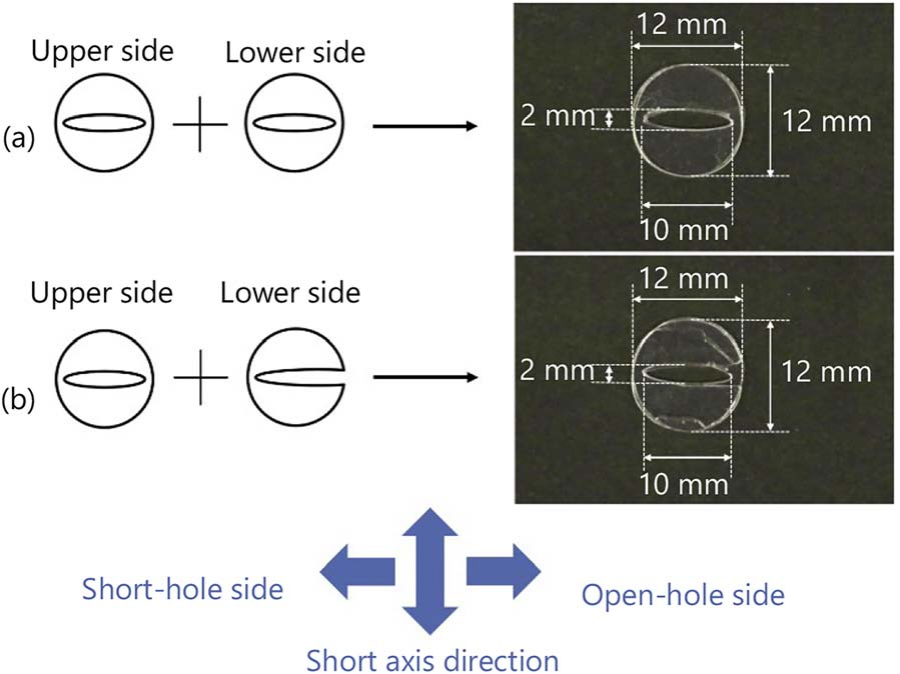
Exoskeleton frames used in Experiment 1. (a) The exoskeleton having a symmetrical hole. (b) The exoskeleton having an asymmetrical hole.

We fabricate two exoskeletons to fix the droplet shape as shown in Fig. 6. One is an exoskeleton having a symmetrical hole, named with a symmetrical exoskeleton hereafter, by bonding two OHP films of the same shape having an elliptical hole (Fig. 6(a)). This exoskeleton is the same with the one used in the preliminary experiment. The other one is an exoskeleton having an asymmetrical hole, named with an asymmetrical exoskeleton hereafter, by bonding two differently shaped OHP films (Fig. 6(b)). The upper side film has the same elliptical hole as the symmetrical exoskeleton, and the lower side film has an open hole in one side. By cutting one end edge of the hole in the exoskeleton, the contact area between a droplet and the aqueous solution increases at the end edge, which causes an asymmetry in the diffusion flux around the droplet. The outer diameter of both exoskeletons is 12 mm, and the thickness is 0.20 mm. The arrows at the bottom of Fig. 6 indicate the directions relative to the asymmetrical exoskeleton.

We drop a 6 μL 1-pentanol droplet into both exoskeletons. The droplet does not overflow through each hole in the exoskeletons, and both droplets are shaped as shown in Fig. 2.

In Experiment 1, we observe the behavior of the self-propelled droplet dropped into an exoskeleton frame, and measured the velocity of the self-propulsion. The experiments are conducted by using the same setup used in the preliminary experiment, as shown in Fig. 3. As described in the preliminary experiment section, we use two aqueous solutions of different concentrations: one with 0.5 mL/100 mL water and one with 2.3 mL/100 mL water. Therefore, experiments are conducted for four conditions, combining the two different concentrations of aqueous solution and the two different exoskeletons.

#### 4.1.2 Result and discussion

**Fig. 7:**
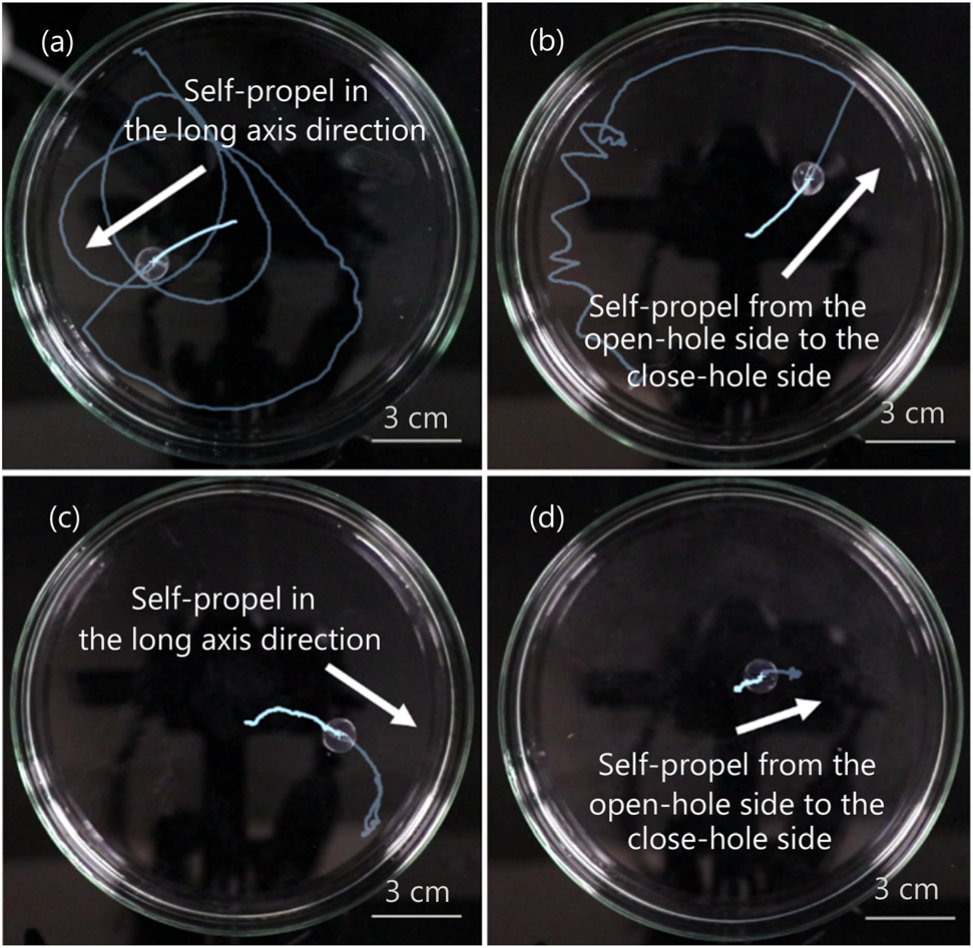
The behavior of the self-propelled droplet. (a) 0.5 mL/100 mL water, the symmetrical exoskeleton. (b) 0.5 mL/100 mL water, the asymmetrical exoskeleton. (c) 2.3 mL/100 mL water, the symmetrical exoskeleton. (d) 2.3 mL/100 mL water, the asymmetrical exoskeleton.

The behaviors of the self-propelled 1-pentanol droplets are shown in Fig. 7 and Movie S1–S4.† In the case of using the symmetrical exoskeleton (Fig. 7(a) and (c)), the droplet was self-propelled in the long axis direction. On the other hand, in the case of the asymmetrical exoskeleton (Fig. 7(b) and (d)), all the droplets were self-propelled in the direction from the open-hole side to the close-hole side.

Gidituri *et al.* constructed the model of the Marangoni surfer using the advection–diffusion equation.^21^ According to the results of the numerical simulations, when the droplet shape is a thin rod and releases the surfactant from the center, the trajectory of the droplet becomes a straight line. Furthermore, when the droplet shape is a thin rod and releases the surfactant from one of its ends, the droplet self-propels straightly using the surfactant acting as a propulsion engine. These results are consistent with the trajectories of the self-propelled 1-pentanol droplet.

**Fig. 8:**
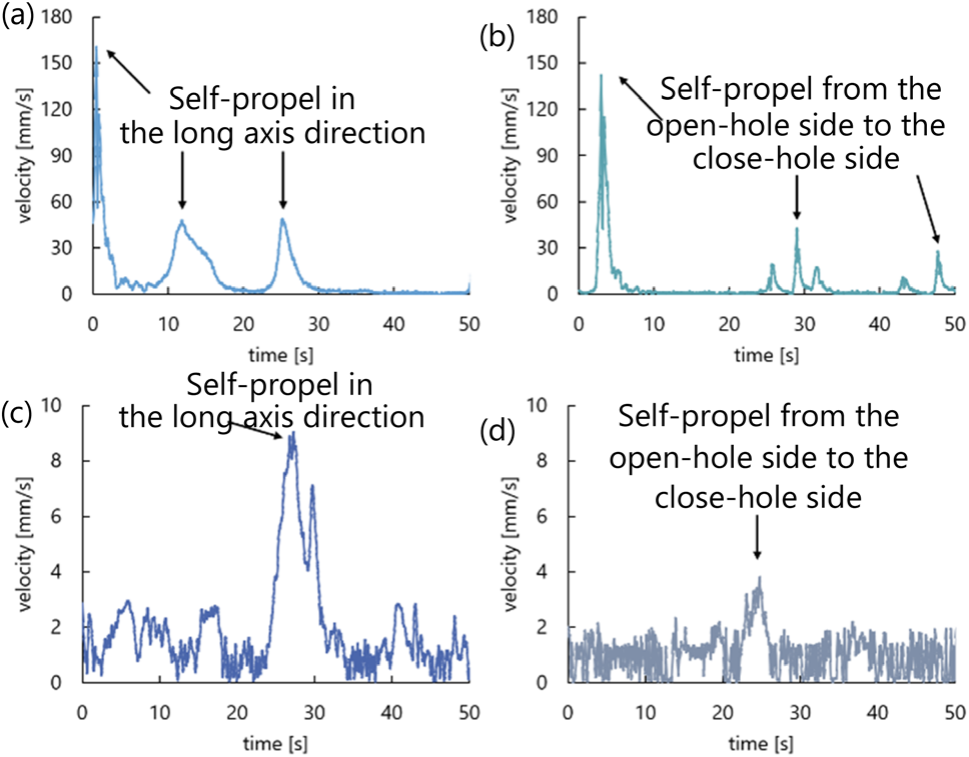
Time evolution of the self-propulsion velocity. (a) 0.5 mL/100 mL water, the symmetrical exoskeleton. (b) 0.5 mL/100 mL water, the asymmetrical exoskeleton. (c) 2.3 mL/100 mL water, the symmetrical exoskeleton. (d) 2.3 mL/100 mL water, the asymmetrical exoskeleton.

The time evolution of the velocity of the self-propelled droplets is shown in Fig. 8. When the droplets were self-propelled in the long axis direction or the direction from the open-hole side to the close-hole side, the self-propulsion velocity temporarily increased. When the aqueous solution with 0.5 mL/100 mL water was used (Fig. 8(a) and (b)), the self-propulsion in the long axis direction or the direction from the open-hole side to the close-hole side was observed several times in 50 seconds.

**Fig. 9:**
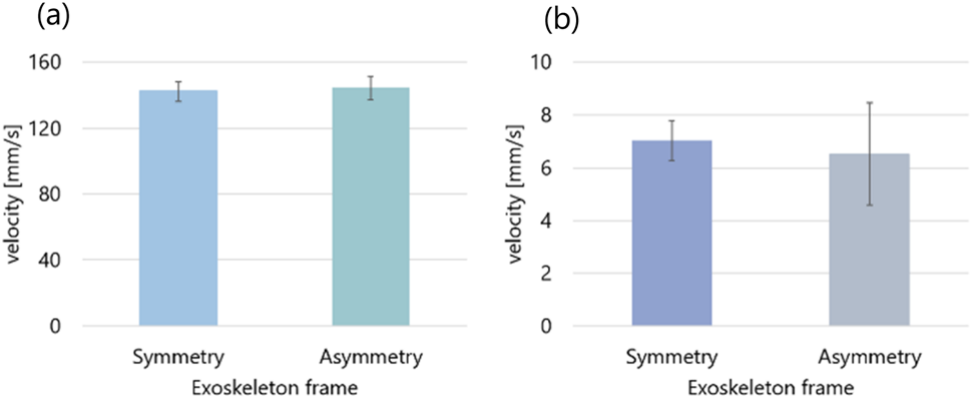
Relationship between the shape of the exoskeletons and average velocity. (a) The concentration of the aqueous solution is 0.5 mL/100 mL water. (b) The concentration of the aqueous solution is 2.3 mL/100 mL water.

We calculated the average and standard deviation of the maximum velocities from the results of five experiments in each of the four conditions. Fig. 9 shows the calculation results. The droplet was self-propelled faster in the case of the aqueous solution with 0.5 mL/100 mL water (Fig. 9(a)) than in the case of the aqueous solution with 2.3 mL/100 mL water (Fig. 9(b)), which was the same as the results of the preliminary experiment.

According to Fig. 9(a), the average of the maximum velocities of the self-propelled droplets dropped into the symmetric exoskeleton was 1.42 × 10^2^ mm s^−1^, and that into the asymmetric exoskeleton was 1.44 × 10^2^ mm s^−1^.

Furthermore, according to Fig. 9(b), the average of the maximum velocities of the self-propelled droplets dropped into the symmetric exoskeleton was 7.03 mm s^−1^, and that into the asymmetric exoskeleton was 6.51 mm s^−1^. The self-propulsion velocity of the droplets did not change significantly between the case with the symmetrical exoskeleton and with the asymmetrical exoskeleton. We found that the droplet could determine the direction of self-propulsion without losing velocity by using the asymmetrical exoskeleton.

### 4.2 Experiment 2: observation of convection

#### 4.2.1 Experimental method

In Experiment 2, we conduct the three-dimensional observation of the Marangoni convection around and inside a 1-pentanol droplet. Three-dimensional convection flow is generated around and inside the droplet. We observe the convection from two directions to investigate its characteristics, and analyzed the convection using particle image velocimetry (PIV). To visualize the convection, we mix acrylic particles (Lumisis marker, specific gravity 1.3, Stokes number *S* ≪ 1) with a droplet and an aqueous solution, and observe the flow under ultraviolet illumination. These particles fluoresce under ultraviolet light to visualize the flow of fluid.

To fix the droplet location in the aqueous solution, we fabricate two types of an OHP film. Since we use a rectangular Petri dish in this experiment, the OHP films are designed to be rectangular so that they cover the surface of the aqueous solution introduced into the Petri dish. These OHP films have holes of the same shape with the hole in the exoskeleton shown in Fig. 6. The hole for dropping the droplet is placed in a position where the convection inside the droplet and the aqueous solution can be observed and where the convection is not affected by the walls of the Petri dish.

**Fig. 10:**
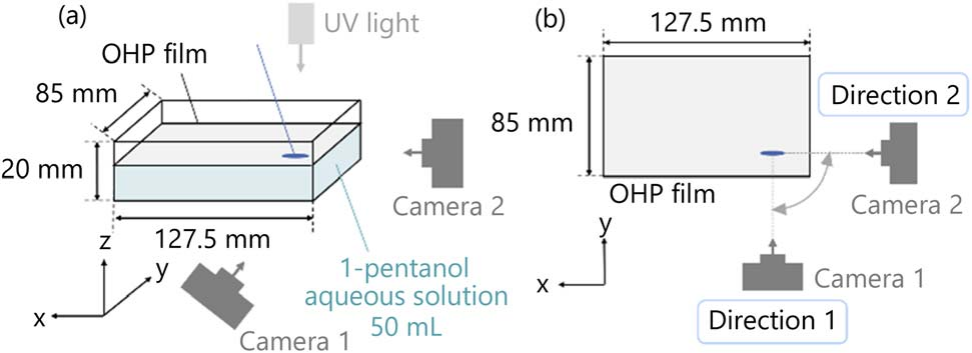
Experimental setup of Experiment 2. (a) Schematic diagram of the experimental setup. (b) The positional relationship between the droplet and the cameras from the top view of the experimental setup.

The experimental setup is shown in Fig. 10(a). After filling 50 mL of 1-pentanol aqueous solution in a Petri dish of rectangular shape, we float the OHP film to cover the surface of the aqueous solution. Then, an automatic micropipette introduces a 12 μL of 1-pentanol droplet in the hole of the OHP film. The droplet volume is doubled from Experiment 1 to increase the cross-sectional area of the droplet for easily observing the convection inside the droplet. The droplet does not overflow from the hole in the exoskeleton, and their length in the depth direction increases. The convection is captured from two directions (Fig. 10(b)) using two high-speed cameras (Baumer, VCXU-13M, VCXU.2–13M). The direction to observe the convection in the *z*–*x* plane is defined as Direction 1, and the movies are taken by Camera 1. Similarly, the direction to observe the convection in the *y*–*z* plane is defined as Direction 2, and the movies are taken by Camera 2. The movies are analyzed using FlowExpert2D2C software. These experiments are performed on an experimental desk placed horizontally. The temperature is about 25 °C and the humidity is about 60%.

#### 4.2.2 Result and discussion about the convection around the droplet

**Fig. 11:**
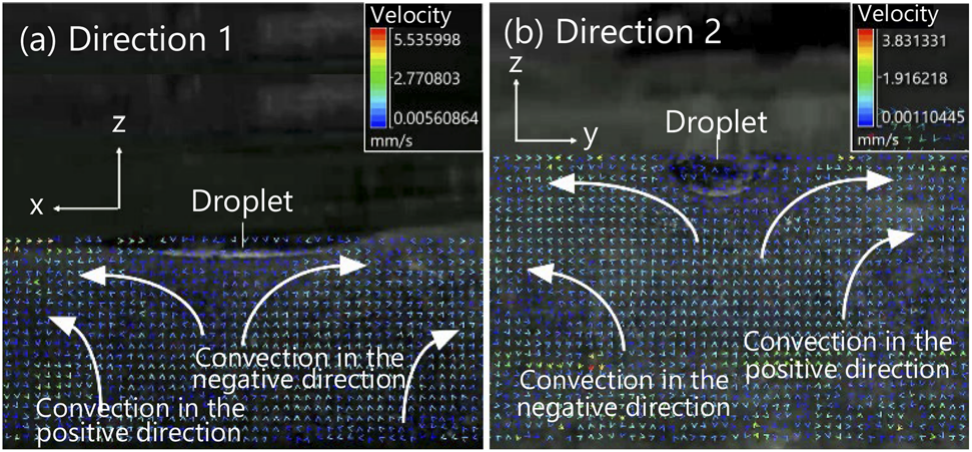
Convection generated around a droplet, under the condition of using the aqueous solution with a concentration of 0.5 mL/100 mL of water and the symmetrical exoskeleton. (a) Convection captured from Direction 1. (b) Convection captured from Direction 2.

Firstly, we analyzed the convection generated in the aqueous solution around the droplet. We observed a pair of convection generated in the positive and negative direction relative to the droplet in all for experimental conditions. Fig. 11 shows the PIV data in the case of using the aqueous solution with a concentration of 0.5 mL/100 mL water and the symmetrical exoskeleton. PIV data for other conditions is shown in Fig. S1–S3 in the ESI.†

The convections appeared between 80 and 140 seconds after a droplet being dropped into the aqueous solution in all four conditions. The rising of convection was slower than the cases of the observation in Experiment 1 (Fig. 8). This is because the surface of the aqueous solution was covered with OHP film in Experiment 2. When the 1-pentanol aqueous solution is exposed to air, the evaporation of 1-pentanol around the solution's surface is greater to generate the greater surface tension gradient, which induces the convection inside the solution faster.^40^ In the case of Experiment 2, the surface of the aqueous solution was covered with OHP film, and the surface tension initially stayed static to delay the start of generating the tension gradient. Without considering the evaporation of 1-pentanol, convection caused by the difference in concentration between the droplet and the aqueous solution was slower to appear.

**Fig. 12:**
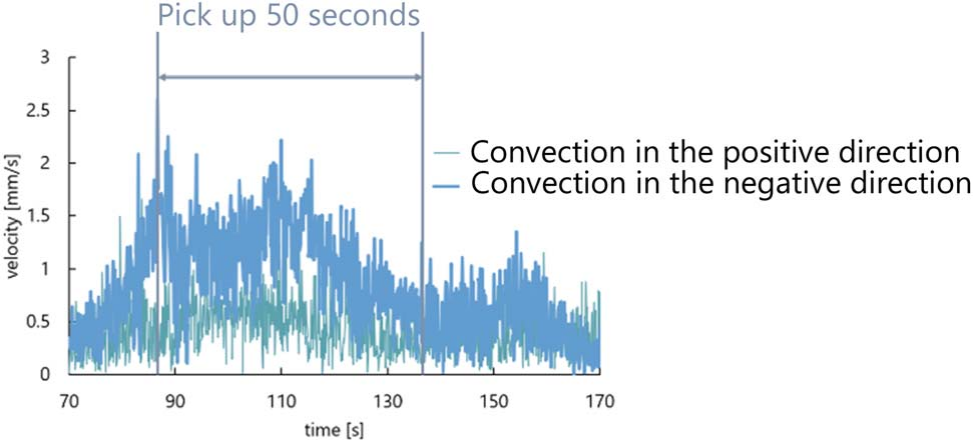
Time evolution of the convection velocity generated in the positive and negative direction relative to the droplet. This result was the condition of using the aqueous solution with a concentration of 0.5 mL/100 mL of water and the symmetrical exoskeleton.

We measured the velocity of convection generated on both sides of the droplet under each condition. The velocity of the convection starts increasing when the convection is initially generated. Herein, we extracted 50 seconds from the time of the maximum velocity of convection observed as shown in Fig. 12. Tables 1 and 2 show the results of calculating the average and the standard deviation of the convection velocity during this period. The observational results from Direction 1 show a difference in the velocity of the convection generated in the positive and negative direction relative to the droplet in all conditions. On the other hand, according to the observational results from Direction 2, the convection velocity generated in the positive and negative direction was almost the same for all conditions.

**Table 1:** Average and standard deviation of the velocity of the convection around the droplet observed from Direction 1

Concentration	Exoskeleton	Positive direction		Negative direction	
		Velocity	Standard deviation	Velocity	Standard deviation
0.5 mL/100 mL water	Symmetrical	0.476	0.248	1.154	0.457
	Asymmetrical	0.347	0.181	0.708	0.268
2.3 mL/100 mL water	Symmetrical	1.407	0.998	0.816	0.590
	Asymmetrical	0.320	0.215	0.605	0.313

**Table 2:** Average and standard deviation of the velocity of the convection around the droplet observed from Direction 2

Concentration	Exoskeleton	Negative direction		Positive direction	
		Velocity	Standard deviation	Velocity	Standard deviation
0.5 mL/100 mL water	Symmetrical	0.850	0.262	0.558	0.200
	Asymmetrical	0.416	0.258	0.505	0.198
2.3 mL/100 mL water	Symmetrical	0.454	0.253	0.320	0.178
	Asymmetrical	0.209	0.136	0.310	0.139

**Fig. 13:**
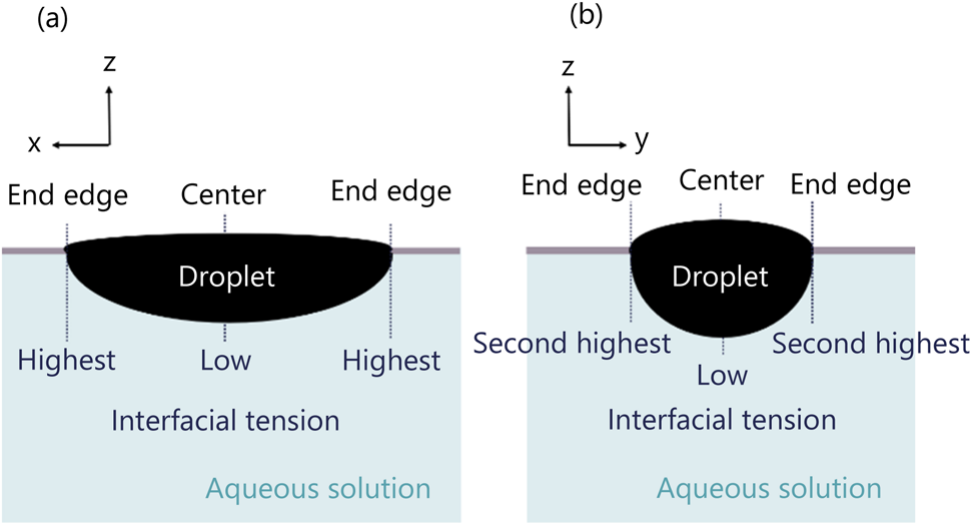
Distribution of interfacial tension of droplet. (a) Droplet observed from Direction 1. (b) Droplet observed from Direction 2.

The convection around the droplet is generated by the diffusion of 1-pentanol from the droplet into the aqueous solution. A droplet is fixed in an elliptical shape by being dropped into the exoskeleton hole. The curvature of the droplet shape causes the interfacial tension difference as presented by eqn (2). As shown in Fig. 13, the interfacial tension decreases in the following order; the end edge of the droplet observed from Direction 1, the end edge of the droplet observed from Direction 2, and the center of the droplet. This difference in interfacial tension causes a change in diffusion. This is because lower interfacial tension lowers the energy barrier for molecular exchange between phases, and lower interfacial tension lowers diffusion. This interfacial tension difference induces convection around the droplet.

**Fig. 14:**
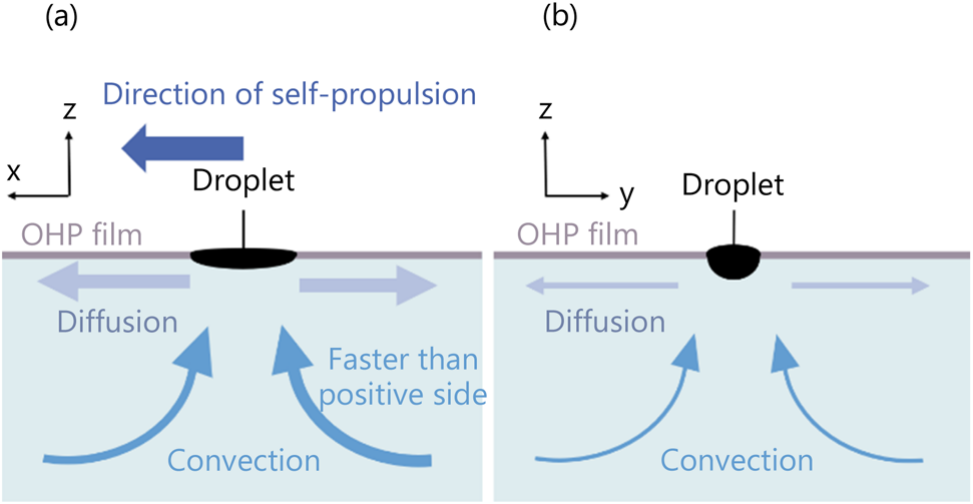
Schematic diagram of convection generation around the droplet. (a) Convection observed from Direction 1 when the droplet self-propels in the positive direction. (b) Convection observed from Direction 2.

Firstly, we study the convection observed from Direction 1 (Fig. 14(a)). When the symmetrical exoskeleton is used, a slight asymmetry between the positive-side diffusion and the negative-side diffusion generates the different velocities of the convection in the positive and negative sides observed from Direction 1. The convection with the higher velocity pushes the droplet to be self-propelled to the opposite side of the convection. Since the diffusion asymmetry is spontaneously generated, the self-propulsion occurs in the long axis direction.

When an asymmetrical exoskeleton is used, the open-hole side end edge of the droplet has a larger contact area between the droplet and the aqueous solution. The diffusion flux is greater around the open-hole side end edge, which generates greater convection. As shown in Table 1, all the self-propulsion directions are determined by the open-hole side end edge, and the asymmetry of the exoskeleton controls the self-propulsion by the effect of convection induced by using an asymmetrical exoskeleton.

In the observation from Direction 2, the diffusion of 1-pentanol causes symmetric convection (Fig. 14(b)). The interfacial tension at the center of the droplet is smaller than at the end edges due to the curvature of the droplet shape,^36^ so the diffusion of 1-pentanol is less at the center. Therefore, the velocity of convection is also smaller at the center. A slight asymmetry occurs in the convection generated in the positive and negative sides of the droplet, however, it does not affect the self-propulsion direction because the difference between the positive and negative sides of the convection observed from Direction 1 is greater than the convection observed from Direction 2.

#### 4.2.3 Result and discussion about the convection inside the droplet

**Fig. 15:**
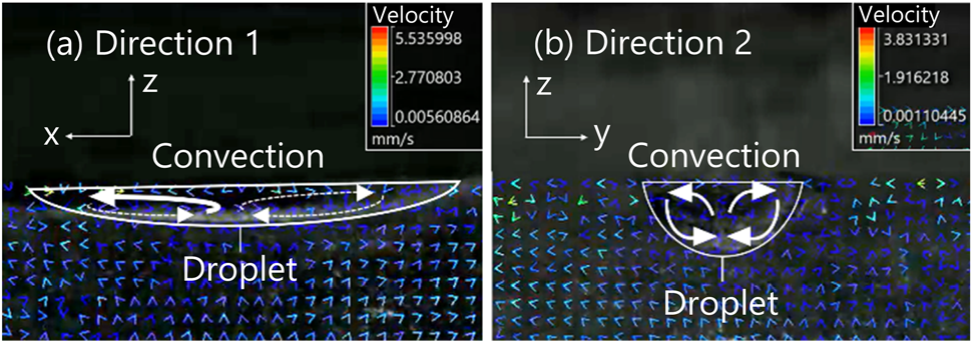
Convection generated inside a droplet, under the condition of using the aqueous solution with a concentration of 0.5 mL/100 mL of water and the symmetrical exoskeleton. (a) Convection captured from Direction 1. (b) Convection captured from Direction 2.

We analyzed the convection generated inside the droplet. We observed a pair of convection in the positive and negative direction relative to the droplet from Direction 1, and the one-side convection is greater. The greater convection is indicated by solid arrows in the figures. From Direction 2, a pair of convection which have mostly the same size was observed. Fig. 15 shows the PIV data in the case of using the aqueous solution with a concentration of 0.5 mL/100 mL water and the symmetrical exoskeleton. PIV data for other conditions is shown in Fig. S4–S6 in the ESI.†

**Fig. 16:**
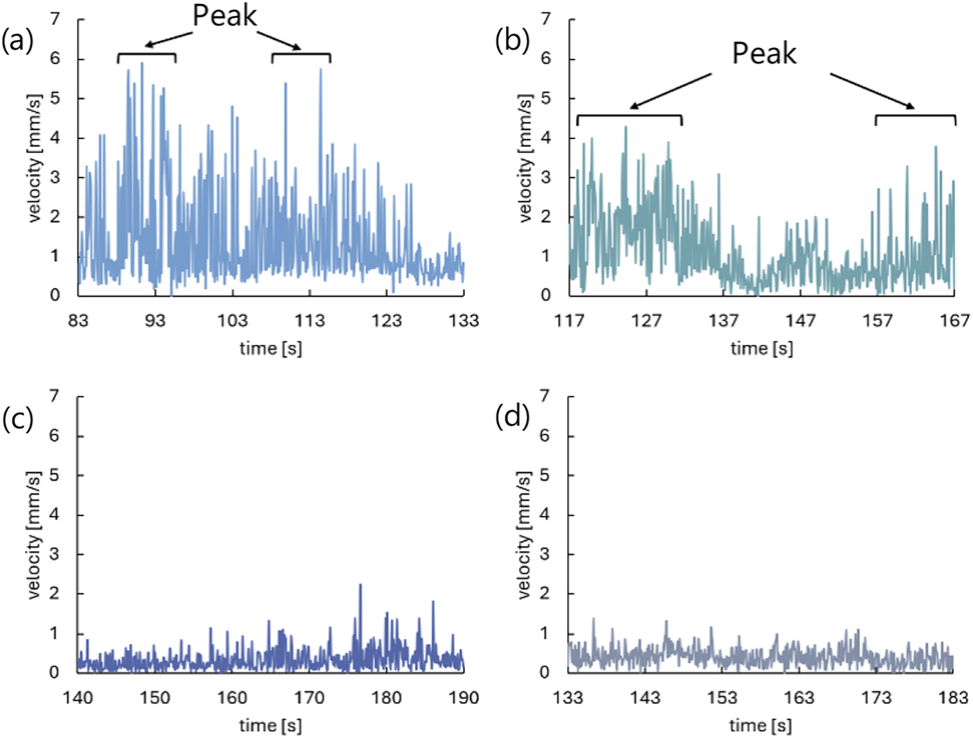
Time evolution of convection velocity of the fast side observed from Direction 1 in 50 seconds extracted for each condition. (a) 0.5 mL/100 mL water, the symmetrical exoskeleton. (b) 0.5 mL/100 mL water, the asymmetrical exoskeleton. (c) 2.3 mL/100 mL water, the symmetrical exoskeleton. (d) 2.3 mL/100 mL water, the asymmetrical exoskeleton.

We measured the convection velocity generated inside the droplet under each condition and observational direction. The same 50 seconds as being conducted in the convection analysis around the droplet were used to calculate the average and the standard deviation of the convection velocity during this period. The results are shown in Tables 3 and 4. The observational results from Direction 1 indicate that the velocity of generated convection depends on the concentration of the aqueous solution. When the aqueous solution with 0.5 mL/100 mL water was used, we found the convection generated with the velocity of more than three times the average (Fig. 16). On the other hand, according to the observational results from Direction 2, the convection velocity generated in the positive and negative sides relative to the droplet was almost the same for all conditions.

**Table 3:** Average and standard deviation of the velocity of the convection inside the droplet observed from Direction 1

Concentration	Exoskeleton	Positive direction		Negative direction	
		Velocity	Standard deviation	Velocity	Standard deviation
0.5 mL/100 mL water	Symmetrical	1.389	1.116	0.633	0.388
	Asymmetrical	1.077	0.848	0.402	0.242
2.3 mL/100 mL water	Symmetrical	0.185	0.119	0.339	0.279
	Asymmetrical	0.420	0.226	0.312	0.171

**Table 4:** Average and standard deviation of the velocity of the convection inside the droplet observed from Direction 2

Concentration	Exoskeleton	Negative direction		Positive direction	
		Velocity	Standard deviation	Velocity	Standard deviation
0.5 mL/100 mL water	Symmetrical	0.173	0.115	0.160	0.092
	Asymmetrical	0.240	0.179	0.122	0.280
2.3 mL/100 mL water	Symmetrical	0.392	0.289	0.243	0.237
	Asymmetrical	0.642	0.468	0.623	0.359

Firstly, we consider the shape of the convection generated inside the droplet. The convection inside the droplet is generated by the interfacial tension of the droplet. The interfacial tension is distributed as shown in Fig. 13, and the convection is generated accordingly in the direction from the lower to the higher interfacial tension.

**Fig. 17:**
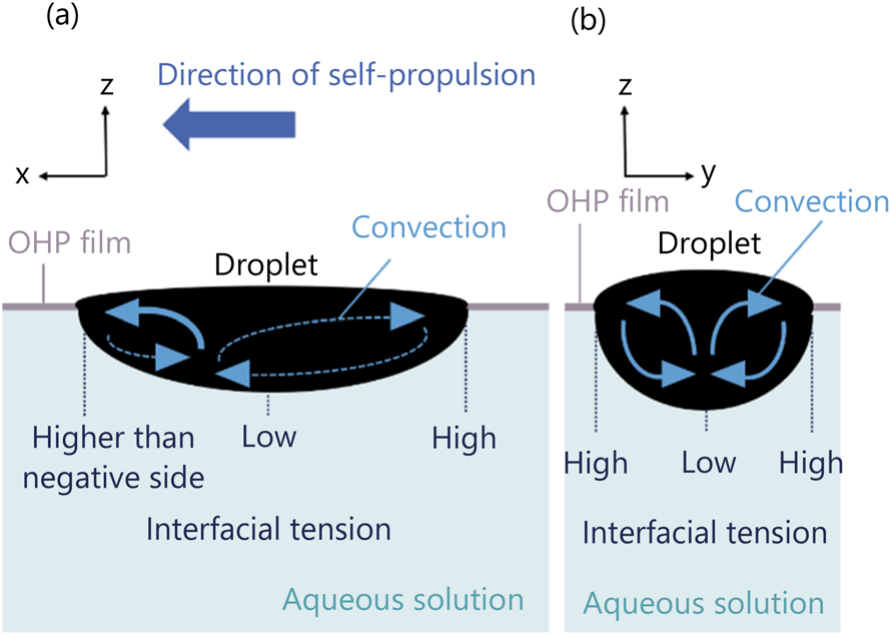
Schematic diagram of convection generation inside the droplet. (a) Convection observed from Direction 1 when the droplet self-propels in the positive direction. (b) Convection observed from Direction 2.

We discuss the convection observed from Direction 1 (Fig. 17(a)). When a symmetrical exoskeleton is applied, a slight asymmetry occurs between the diffusion from the positive and negative sides' end edges, so that a slight difference in the concentration gradient is generated (|d*c*/d*x*|_positive side edge_| > |d*c*/d*x*|_negative side edge_| or |d*c*/d*x*|_positive side edge_| < |d*c*/d*x*|_negative side edge_|). Then the slight difference in the interfacial tension is also generated. Here, we define the interfacial tension difference between the positive side end edge and the center of the droplet as *γ*_positive side_, and that between the negative side end edge and the center of the droplet as *γ*_negative side_, the relation between *γ*_positive side_ and *γ*_negative side_ is described as *γ*_positive side_ > *γ*_negative side_ or *γ*_positive side_ < *γ*_negative side_, according to eqn (2). Therefore, the convection is strongly generated on the side with the greater difference in the interfacial tension. The generated convection and the self-propulsion direction of the droplet are observed in the same direction.

When the asymmetrical exoskeleton is applied, the contact area between the droplet and the aqueous solution is larger at the negative side end edge of the droplet, so the diffusion is greater at the negative side end edge. Therefore, the 1-pentanol concentration gradient of the aqueous solution around the negative side end edge is smaller than that around the positive side end edge (|d*c*/d*x*|_positive side edge_| > |d*c*/d*x*|_negative side edge_), and the interfacial tension difference between the negative side end edge and the center of the droplet is also smaller than that between the positive side end edge and the center of the droplet (*γ*_positive side_ > *γ*_negative side_). Therefore, interfacial tension is intentionally stronger at the positive side end edge by using the asymmetrical exoskeleton, and convection is generated in the positive direction.

The interfacial tension of the 1-pentanol droplet generated two convections, which were observed as the symmetrical flow from Direction 2 (Fig. 17(b)). Due to the droplet shape, the interfacial tension difference observed from Direction 2 is smaller than that from Direction 1. Therefore, the velocity of the generated convection is comparatively slow and does not affect the self-propulsion direction and velocity.

Next, we consider the cause of the difference in the convection velocity depending on the aqueous solution concentration in the observation from Direction 1. As discussed in the Preliminary experiment, the smaller the aqueous solution concentration is, the greater the difference in interfacial tension between the end edge and the center of the droplet becomes. Therefore, the convection inside the droplet is faster when the aqueous solution with 0.5 mL/100 mL water is used than when the aqueous solution with 2.3 mL/100 mL water is used.

In Experiment 1 to observe the behavior of the self-propelled droplet, the droplet was self-propelled discretely several times during 50 seconds when the aqueous solution with 0.5 mL/100 mL water was used (Fig. 8(a) and (b)). The velocity of the droplet presented several peaks in time in Experiment 2 (Fig. 16). These results suggest that the convection inside the droplet is closely related to the self-propulsion velocity and the number of self-propulsion times. The droplet repeats self-propulsion several times because the convection is generated around and inside the droplet again at the location where it moved after the self-propulsion.

### 4.3 Summarization of total analysis

We investigated the characteristics of three-dimensional convection generated around and inside the droplet by observing convection from the two directions. The convection around the droplet is generated by the diffusion of 1-pentanol from the droplet into the aqueous solution. The difference in diffusion between the positive and negative sides' end edges relative to the droplet causes the difference in convection velocity, and the fast convection pushes the droplet to promote self-propulsion (Fig. 14(a)). The convection inside the droplet, on the other hand, is generated by the interfacial tension of the droplet. The difference in interfacial tension depends on the difference in the diffusion flux between the droplet's positive and negative sides' end edges. The convection is generated in the direction of strong interfacial tension, which coincides with the self-propulsion direction of the droplet (Fig. 17(a)). The self-propulsion of a droplet is caused by the superposition of convections around and inside the droplet.

## 5 Conclusions

In this study, we observed convection generated three-dimensionally around and inside a self-propelled 1-pentanol droplet from two directions. We analyzed the relationship between the droplet's self-propulsion behavior and the convection flow. The experiment used two aqueous solutions of different concentrations and two exoskeletons with different hole shapes. The exoskeletons were fabricated by bonding two OHP films together, which were the symmetrically-shaped exoskeleton and the asymmetrically-shaped exoskeleton.

When the droplet is dropped into the symmetrical exoskeleton, the droplet is self-propelled in the long axis direction. When the asymmetrical exoskeleton is used, the self-propulsion direction of the droplet is defined in the direction from the open-hole side to the close-hole side. The smaller the aqueous solution concentration is, the faster the self-propulsion velocity becomes.

The convection generated around the droplet is caused by the diffusion of 1-pentanol from the droplet to the aqueous solution. The observation from Direction 1 indicates that there is a difference in the velocity of the convection generated in the positive and negative sides of the droplet. The convection with high velocity pushes the droplet, so that the droplet is self-propelled to the opposite side of the convection with high velocity.

The convection generated inside the droplet is caused by the interfacial tension of the droplet. The observation from Direction 1 indicates that convection is generated strongly in the same direction as the droplet's self-propulsion direction. The velocity of this convection increases with decreasing the aqueous solution concentration.

In the next study, we will investigate the optimal conditions for controlling self-propelled droplets. Since the self-propulsion of the 1-pentanol droplet examined in this study was only a linear motion, other motions such as rotational motion and temporary and spatially controlled motions will be investigated by applying differently-shaped exoskeletons. The advantage of self-propulsion is that it does not require any external energy supply, such as electricity, and moves autonomously using chemical energy extracted from chemical reactions. By clarifying the mechanism of self-propelled droplets, the self-propulsion will be applied as a driving source for soft robots that move only by chemical reactions.

## Data availability

The data supporting this article have been included as part of the ESI.†

## Author contributions

Tamako Suzuki performed all the experiments and wrote the manuscript. Hideyuki Sawada directed and supervised the research project and wrote the manuscript. All the authors discussed the results.

## Conflicts of interest

There are no conflicts to declare.
